# Resveratrol-Enriched *Polygonum cuspidatum* Extract Enhances Functional Bioactivity Against Non-Small Cell Lung Cancer Through Modulation of Inflammatory Signaling and Mitochondrial Apoptosis

**DOI:** 10.3390/nu18121862

**Published:** 2026-06-09

**Authors:** Ho-Lin Wang, Hui-Pei Huang, Naveen Ranasinghe, Yu-Hsien Lin, Hsiao-Ping Kuo, Shyue-Tsong Huang, Li-Chan Yang, Tai-Lin Chen, Ming-Hon Hou

**Affiliations:** 1 Doctoral Program in Medical Biotechnology, National Chung Hsing University, Taichung 402, Taiwan; 2 Department of Medical Research, Chung Shan Medical University Hospital, Taichung 402, Taiwan; 3 Department of Biochemistry, School of Medicine, Chung Shan Medical University, Taichung 402, Taiwan; 4 Department of Post-Baccalaureate Medicine, College of Medicine, National Chung Hsing University, Taichung 402, Taiwan; madhumalranasinghe@gmail.com (N.R.);; 5 Doctoral Program in Tissue Engineering and Regenerative Medicine, National Chung Hsing University, Taichung 402, Taiwan; 6 Bioresources Collection and Research Center, Food Industry Research and Development Institute, Hsinchu 300197, Taiwan; 7 Graduate Institute of Chinese Medicine and Drug Development, School of Medicine, National Chung Hsing University, Taichung 402, Taiwan; 8 Graduate Institute of Genomics and Bioinformatics, National Chung Hsing University, Taichung 402, Taiwan; 9 Biotechnology Center, National Chung Hsing University, Taichung 402, Taiwan

**Keywords:** *Polygonum cuspidatum*, biotransformation, resveratrol, NSCLC, apoptosis, inflammasome

## Abstract

**Background:** *Polygonum cuspidatum* (Hu Zhang) is a polyphenol-rich botanical in which resveratrol occurs at low levels mainly as polydatin. In this study, we generated a microbially converted *P. cuspidatum* extract (CPE) with markedly enriched resveratrol content from the unconverted *P. cuspidatum* extract (PE), and evaluated its anticancer efficacy and safety in comparison with those of the unconverted extract. **Methods:** High-performance liquid chromatography (HPLC), A549 cell-based assays, an A549 xenograft model, and a 28-day repeated-dose rat study were performed. **Results:** HPLC showed near-complete depletion of polydatin, marked enrichment of resveratrol, and a modest reduction in emodin. CPE more effectively inhibited A549 cell proliferation and migration than PE, suppressed NLRP3 inflammasome and nuclear factor kappa B (NFκB) signaling, and promoted reactive oxygen species (ROS) accumulation and mitochondrial apoptosis. Oral CPE also significantly reduced tumor growth in an A549 xenograft model. In a 28-day repeated-dose rat study, no treatment-related abnormalities were observed in blood biochemistry or histopathology. **Conclusions:** These findings support CPE as a promising functional food ingredient.

## 1. Introduction

Non-small-cell lung cancer (NSCLC) is a heterogeneous group of tumors that accounts for approximately 85% of all lung cancer cases and remains the leading cause of cancer-related mortality worldwide [1]. In addition to tobacco smoking, environmental factors such as radon exposure and air pollution contribute significantly to NSCLC incidence, underscoring the complex etiology of this disease [2]. Over the past decade, the introduction of low-dose computed tomography screening, advances in surgical and radiotherapeutic techniques, and the development of targeted and immune-based therapies have improved early detection and clinical management of NSCLC [2,3]. Nevertheless, long-term survival remains limited for many patients due to therapeutic resistance, cumulative toxicity, and frequent disease recurrence. Together, these challenges highlight the ongoing need to identify new therapeutic strategies that can improve efficacy while minimizing adverse effects.

*Polygonum cuspidatum*, commonly known as Japanese knotweed and referred to as Hu Zhang in traditional Chinese medicine, has been widely used for its anti-inflammatory, antioxidant, and cardioprotective properties [4,5,6]. Among the various bioactive constituents of *P. cuspidatum*, resveratrol is considered the most valuable compound, and the rhizome of this plant is particularly rich in stilbene derivatives, including resveratrol and its glycoside polydatin [7,8]. Among these, resveratrol has attracted substantial attention due to its reported anticancer activities [9], which include inhibition of cell proliferation, induction of apoptosis, modulation of inflammatory signaling, and regulation of oxidative stress [10,11,12].

Beyond its diverse pharmacological effects, resveratrol is widely used in the food additive, nutraceutical, and cosmetic industries [13,14] and has been reported to reduce the risk of cardiovascular disease [15] and inhibit proliferation in multiple cancer cell types [16]. However, current production relies primarily on plant extraction, plant cell culture, or recombinant microbial biosynthesis [17]. These approaches are often constrained by high costs and technical complexity, highlighting the need for more efficient and economically viable production strategies [10,18,19].

Therefore, the clinical and experimental utility of resveratrol is limited by its low natural abundance in plant materials and poor bioavailability. In *P. cuspidatum*, resveratrol predominantly exists as its glycosylated form, polydatin, which exhibits lower biological activity than the aglycone. Strategies that increase resveratrol content while preserving the natural plant matrix could improve anticancer efficacy and provide reliable platforms for production and extraction. Such approaches may also reduce dependence on imported raw materials, support the development of resveratrol-based health products, and expand their commercial applications [20,21,22]. Despite these advantages, polydatin’s glycosylated structure limits its bioavailability. Conventional methods for converting polydatin into resveratrol, including acid- or alkali-mediated hydrolysis, require harsh conditions, such as elevated temperature and pressure, which impose equipment constraints and increase the environmental burden [23]. These limitations underscore the need for alternative strategies that enhance resveratrol availability while maintaining the integrity of the plant matrix.

To overcome these limitations, we developed a microbial biotransformation process that converts polydatin in crude *P. cuspidatum* material into resveratrol under mild conditions [24,25]. Although the anticancer activities of *P. cuspidatum* extracts have been reported, their efficacy in NSCLC and the potential added value of microbial transformation have not been systematically evaluated. Here, we investigated whether a microbially converted *P. cuspidatum* extract (CPE) provides improved anticancer efficacy and safety compared with the unconverted extract (PE). We first profiled the phytochemical compositions of PE and CPE by HPLC. We then compared their anti-proliferative, anti-migratory, anti-inflammatory, antioxidant, and pro-apoptotic activities in A549 NSCLC cells. Finally, we evaluated in vivo antitumor efficacy using an A549 xenograft mouse model and assessed safety in a 28-day oral toxicity study in rats, including serum biochemistry and histopathological examination of major organs.

## 2. Materials and Methods

### 2.1. Cell Culture

The human NSCLC cell line A549 (ATCC CCL-185; BCRC 60074) was obtained from the Food Industry Research and Development Institute (Hsinchu, Taiwan). Cells were cultured in 75T flasks using F-12K medium (pH 7.3; Gibco, Waltham, MA, USA) supplemented with 10% fetal bovine serum (FBS; Gibco, Ciudad de México, Mexico), 2 mM L-glutamine, 1% antibiotic–antimycotic solution (PSG; Gibco, Grand Island, NY, USA), and 1.5 g/L sodium bicarbonate (Sigma-Aldrich, St. Louis, MO, USA). Cultures were maintained at 37 °C in a humidified incubator with 5% CO_2_.

For subculturing, cell confluence and morphology were examined under an inverted phase-contrast microscope (CKX41SF, Olympus, Tokyo, Japan). Adherent cells were rinsed twice with phosphate-buffered saline (PBS) and detached using 1 mL of 0.25% trypsin–EDTA solution. After 5 min of incubation, the enzymatic reaction was neutralized by adding an equal volume of complete medium. The cell suspension was collected in a 15 mL conical tube and centrifuged at 1000 rpm for 5 min. The resulting pellet was resuspended in complete medium, and viable cells were counted using a hemocytometer before seeding into plates or dishes as required for each experiment.

PE and CPE were freshly prepared in DMSO. Cells were treated with either PE or CPE at concentrations of 6.25, 12.5, 25, or 50 µg/mL. Control groups received an equivalent volume of vehicle medium.

### 2.2. Microbial Bioconversion of P. cuspidatum

Microbial bioconversion of *P. cuspidatum* was performed following previously reported methods with minor modifications [24,25]. Briefly, the wine yeast *Dekkera bruxellensis*, previously characterized for extracellular β-glucosidase activity and its ability to hydrolyze polydatin/piceid from *P. cuspidatum* into resveratrol, was used as the bioconverting microorganism. The yeast strain was activated in YPD broth at 25 °C with shaking at 150 rpm for 3 days. *P. cuspidatum* powder (2%, *w*/*v*) was suspended in 20 mM acetate buffer (pH 6) and inoculated with the activated *D. bruxellensis* culture at 5% (*v*/*v*). The bioconversion mixture was incubated at 25 °C with shaking at 150 rpm for 2 days. After incubation, samples were collected, freeze-dried, extracted with 80% ethanol, centrifuged at 4000× *g* for 5 min, and filtered through a 0.22 μm membrane prior to HPLC analysis.

### 2.3. Cell Viability Assay (MTT Assay)

Cell viability was assessed using the MTT assay, which measures mitochondrial metabolic activity. A549 cells (2 × 10^4^ cells/well) were seeded into 24-well plates and incubated for 24 h to allow adherence. Cells were then treated with PE or CPE at concentrations of 6.25, 12.5, 25, or 50 μg/mL for 24, 48, and 72 h. After treatment, the media were replaced with fresh medium containing MTT reagent, and the cultures were incubated for 3 h. The medium was removed, and the plates were dried inverted at room temperature. Crystals were dissolved in 1 mL of DMSO, and 100 μL from each well was transferred to a 96-well plate for absorbance measurement at 570 nm using a microplate reader (BioTek, Winooski, VT, USA).

### 2.4. Wound Healing Assay

A549 cells (2 × 10^4^ cells/mL) were seeded in 6-well plates and grown to 90% confluency. The medium was replaced with F-12K containing 0.1% FBS for 24 h to induce cell cycle arrest. A sterile pipette tip was used to scratch the monolayer, creating a wound. Cells were then treated with various concentrations of PE or CPE and incubated for 0–24 h. Wound closure was monitored using phase-contrast microscopy and photographed at designated time points. ImageJ software (1.54j version) was used to quantify the remaining wound area.

### 2.5. Western Blot Analysis

A549 cells (2 × 10^5^ cells/mL) were seeded in 10 cm dishes and cultured for 24 h. The medium was replaced with serum-free F-12K containing 0.1% FBS for 24 h, followed by treatment with PE or CPE for another 24 h. Cells were washed twice with PBS, lysed with RIPA buffer (FIVEphoton Biochemicals, San Diego, CA, USA) containing 1% protease inhibitor cocktail (Roche, Basel, Switzerland), and collected by scraping. Lysates were incubated on a rotating platform at 4 °C for 1 h, then centrifuged at 12,000× *g* for 10 min. Supernatants were collected, and protein concentrations were determined by spectrophotometry. Equal amounts of protein were denatured in loading buffer at 95 °C for 15 min. Samples were stored at −20 °C until analysis.

Proteins were separated by SDS-PAGE and transferred onto PVDF membranes. Membranes were blocked with 5% non-fat dry milk in TBS for 1 h, washed, and incubated with primary antibodies (Table S1) overnight at 4 °C. After washing, membranes were incubated with HRP-conjugated secondary antibodies for 1 h at room temperature. Signals were detected using enhanced chemiluminescence (ECL) reagent, and UV visualized using an imaging system (LAS-4000, GE Healthcare, Tokyo, Japan).

### 2.6. Annexin V Staining Assay

Apoptosis is usually triggered by DNA damage and cell cycle arrest. To confirm the triggering of apoptotic cell death by PE and CPE, we performed an annexin V staining assay. As previously described [26], 1 × 10^6^ A549 NSCLC cells were incubated with PE or CPE alone at various concentrations for 72 h and stained with annexin V-FITC and PI (eBioscience, San Diego, CA, USA) [26]. The stained cells were analyzed by flow cytometry. The treatment of cells with cisplatin was included as a positive control.

### 2.7. Intracellular ROS Measurement

A549 cells in the logarithmic growth phase were seeded at 1 × 10^5^ cells/mL in 96-well plates (200 μL/well) and incubated for 24 h. Cells were then treated with PE or CPE at concentrations of 12.5, 25, 50, 100, or 200 µg/mL for another 24 h. After treatment, the media were removed, and 10 μM DCFH-DA was added to each well. Cells were incubated in the dark for 30 min and then washed with PBS. Fluorescence intensity was measured with excitation at 488 nm and emission at 525 nm using ZEISS Axio Imager 2. DCFH-DA-positive cells were expressed as a percentage of fluorescence intensity relative to the control group.

Relative ROS (%) = (Fluorescence intensity of treated group/Control group) × 100

### 2.8. Animal Study and Xenograft Tumor Model

Animal experiments were conducted according to the guidelines approved by the Institutional Animal Care and Use Committee of National Chung Hsing University (IACUC: 112-136). Male athymic nude mice (5 weeks old) were obtained from the National Center for Biomodels (NCB), NIAR, Taiwan, and housed for 1 week before experimental manipulation. BALB/c nude mice were subcutaneously injected in the right dorsal flank with A549 cells at a density of 1 × 10^7^ cells/mL. Once the tumor volume exceeded 50 mm^3^, mice were randomly assigned to treatment (PE or CPE) and control (normal saline) groups (*n* = 5 per group). The treatment groups received oral gavage of PE or CPE at 300 mg/kg body weight daily for 20 days. The weight and tumor size of the mice were measured twice weekly. Tumor dimensions were measured using a digital caliper, and tumor volume (mm^3^) was calculated using the following formula: length × (width)^2^ × 0.5.

### 2.9. 28-Day Repeated-Dose Oral Toxicity Study of CPE in Male Sprague Dawley Rats

Male Sprague Dawley (SD) rats were purchased from BioLASCO Taiwan Co., Ltd. (Taipei City, Taiwan) and housed under controlled conditions (23 °C; 12 h light/12 h dark cycle) with free access to LabDiet chow (PMI Nutrition LLC, Brentwood, MO, USA) and reverse-osmosis-purified water. CPE were prepared and administered as a suspension in 0.5% (*w*/*v*) carboxymethylcellulose (CMC). For the 28-day repeated-dose oral toxicity study in males, rats (mean body weight ~208 g on the day before dosing) were randomly allocated into four groups (*n* = 10 per group) and gavaged once daily with CPE suspension at 167, or 500 mg/kg, or deionized water (vehicle control), at a fixed dosing volume of 1 mL/100 g body weight [27]. Animals were observed twice daily throughout the dosing period, and body weight was recorded weekly. Prior to termination, 16 h urine samples were collected using metabolic cages for urinalysis. At study end, following overnight fasting, animals were anesthetized with CO_2_, and blood was collected from the abdominal artery for hematology, coagulation, and clinical chemistry analyses. Hematological parameters were measured using an automated hematology analyzer (Sysmex F-800, Sysmex, Kobe, Japan); prothrombin time (PT) and activated partial thromboplastin time (APTT) were determined using a coagulation analyzer (Sysmex CA-1500, Sysmex, Kobe, Japan). Plasma biochemical indices were measured using an automated analyzer (Ciba-Corning 550, Medfield, MA, USA). Urinalysis was performed using reagent strips (N-Multistix SG-L, Ames, Tokyo, Japan). Eyes were examined grossly for abnormalities. At necropsy, major organs (e.g., liver, spleen, kidneys, adrenals, testes/ovaries) were excised, rinsed with ice-cold saline, blotted dry, and weighed; selected tissues (eyes, heart, liver, spleen, kidneys, adrenals, and reproductive tissues) were fixed in 10% neutral buffered formalin for histopathology. Paraffin embedding, sectioning, and hematoxylin and eosin (H&E) staining were performed for the control and high-dose groups; when treatment-related lesions were identified, the corresponding tissues from the low- and mid-dose groups were also processed and examined.

### 2.10. Statistical Analysis

Experiments were performed in triplicate (*n* = 3) unless otherwise indicated. Data are presented as mean ± SEM. Statistical significance was determined by one-way ANOVA followed by Dunnett’s multiple comparisons test (comparing all groups to control). Differences were considered significant at * *p* < 0.05.

## 3. Results and Discussion

### 3.1. HPLC Analysis of Bioactive Compounds in PE and CPE

**Figure 1:**
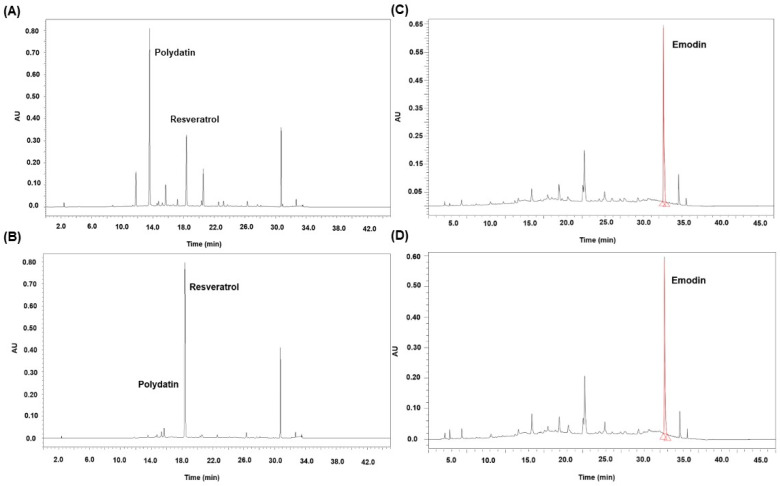
HPLC characterization of major bioactive constituents in *P. cuspidatum* extracts (PE and CPE). Representative HPLC chromatograms of the conventional *P. cuspidatum* extract (PE) and the microbially converted extract (CPE) are shown. Panels (**A**,**B**) depict the polydatin and resveratrol regions for PE and CPE, respectively, whereas panels (**C**,**D**) show the emodin region for PE and CPE, respectively. Peak identities were confirmed by matching retention times with those of authentic reference standards. Polydatin, resveratrol, and emodin eluted at ~13.3 min, 18.1–18.7 min, and ~30.6 min, respectively.

HPLC was used to compare the major bioactive constituents of conventional *P. cuspidatum* extract (PE) and microbially converted *Polygonum cuspidatum* extract (CPE), focusing on polydatin, resveratrol, and emodin. All samples were prepared and analyzed under identical chromatographic conditions, and compound identification was based on retention-time matching to those of authentic standards. In addition, the resveratrol peak in CPE was further supported by HPLC-PDA analysis, which showed a retention time comparable to that of the authentic resveratrol standard (18.249 vs. 18.203 min) and identical UV absorption maxima at approximately 216.2 and 305.2 nm (Figure S1). Distinct and well-resolved peaks corresponding to polydatin (~13 min), resveratrol (~18 min), and emodin (~30 min) were detected in both PE and CPE chromatograms (Figure 1). Quantitative analysis revealed that microbial transformation significantly altered the extract’s chemical composition. The content of polydatin decreased from 7.40 ± 0.07 mg/mL in PE to 0.07 ± 0.02 mg/mL in CPE, while resveratrol content increased from 2.70 ± 0.16 mg/mL to 7.29 ± 0.48 mg/mL. In contrast, emodin content decreased slightly, from 10.34 ± 1.30 mg/mL in PE to 9.16 ± 0.17 mg/mL in CPE.

These results indicate that microbial transformation successfully enriches polyphenolic compounds, particularly resveratrol, while modestly reducing emodin levels. Given the well-documented antioxidant, anti-inflammatory, and anticancer activities of resveratrol, this compositional shift may contribute to the enhanced biological activity observed in CPE. Overall, the HPLC data confirm that microbial transformation is an effective approach for modifying and potentially improving the phytochemical profile of *P. cuspidatum* extracts.

### 3.2. Microbial Transformation Enhances the Anti-Proliferative and Anti-Migratory Effects of P. cuspidatum Extract in A549 Cells

**Figure 2:**
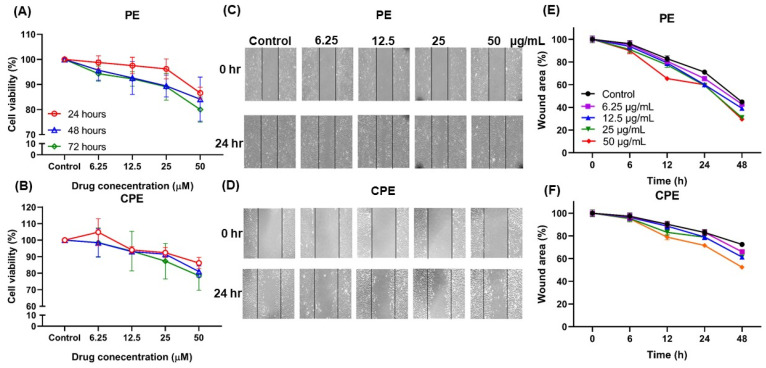
PE and CPE inhibit A549 cell proliferation and migration. A549 cells were treated with PE or CPE (12.5–200 μg/mL) for 0–24 h, and cell viability was assessed by MTT assay. Both extracts reduced viability in a dose- and time-dependent manner, with CPE showing stronger effects (**A**,**B**). Wound-healing assay showing inhibition of A549 cell migration by PE (**C**,**E**) and CPE (**D**,**F**). CPE exhibited greater anti-migratory activity at lower concentrations. Data are mean ± SEM (*n* = 3).

The effects of conventional PE and CPE on A549 NSCLC cells are summarized in Figure 2. Cell viability analysis revealed that both PE and CPE significantly reduced A549 cell viability in a dose- and time-dependent manner at 24, 48, and 72 h post-treatment (Figure 2A,B). Increasing concentrations (6.25–50 μg/mL) resulted in progressive growth inhibition, with more pronounced effects at ≥12.5 μg/mL. Notably, CPE consistently showed slightly stronger and more sustained suppression of cell viability than PE, particularly at 25 and 50 μg/mL, indicating enhanced anti-proliferative activity following microbial transformation.

Representative wound-healing images show that treatment with PE slows wound closure compared with the untreated control (Figure 2C). Quantitative analysis of wound area over time revealed a dose-dependent inhibition of migration, with modest effects observed at 6.25 and 12.5 μg/mL. More pronounced suppression was detected at higher concentrations (25 and 50 μg/mL), particularly at later time points (24–48 h; Figure 2E). These results indicate that PE inhibits A549 cell migration in a concentration- and time-dependent manner, though relatively higher doses are required to achieve strong anti-migratory effects.

Wound-healing reveals a clear, persistent wound gap in CPE-treated cells compared with both the control and PE-treated groups (Figure 2D). Quantitative measurements confirmed a significant reduction in wound closure beginning at 12.5 μg/mL, with progressively greater inhibition at 25 and 50 μg/mL (Figure 2F). Notably, CPE maintained a larger wound area across all time points, indicating a more potent and sustained suppression of cell migration, which microbial transformation enhances the anti-migratory efficacy in A549 lung cancer cells.

### 3.3. PE and CPE Modulate Inflammatory and Oxidative Stress Pathways Relevant to Resveratrol-Mediated Anticancer Activity

**Figure 3:**
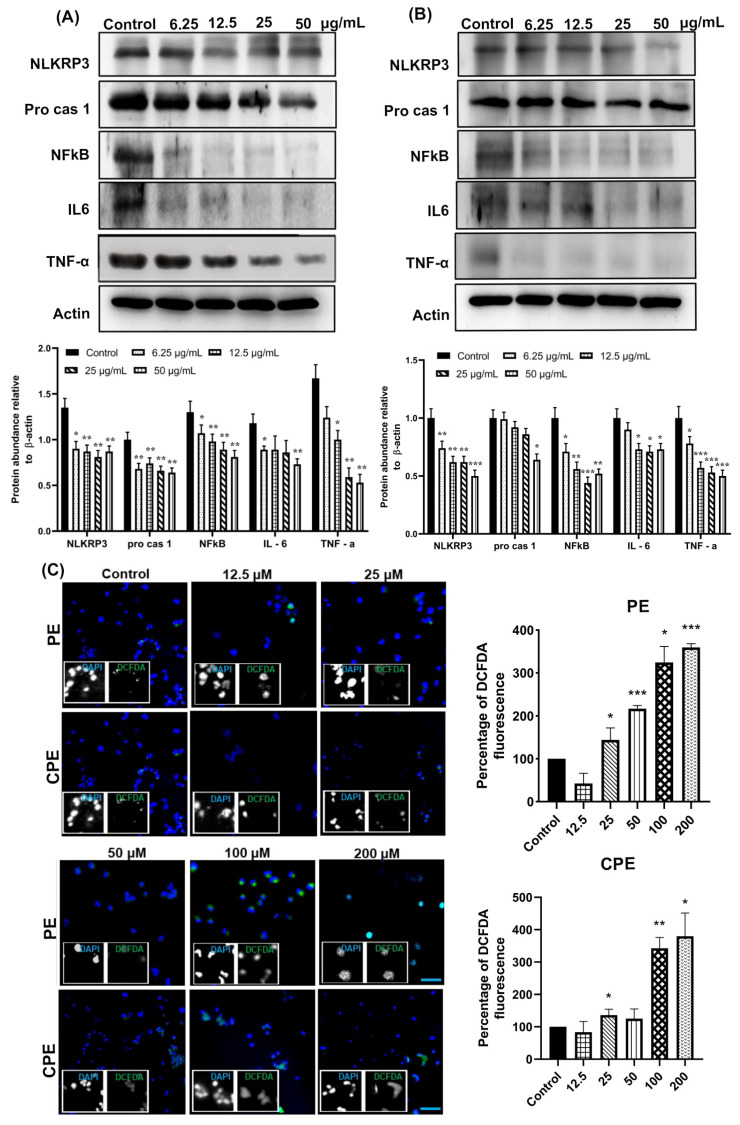
PE and CPE regulate inflammation- and oxidative stress-related pathways in A549 cells. Western blot and densitometric analyses show dose-dependent suppression of NLRP3, pro-caspase-1, NF-κB, IL-6, and TNF-α following PE (**A**) or CPE (**B**) treatment. (**C**) Representative DCFDA fluorescence images and quantification illustrate differential regulation of intracellular ROS, with PE exhibiting antioxidant effects and CPE inducing ROS at higher concentrations. Data are mean ± SEM (*n* = 3). * *p* < 0.05, ** *p* < 0.01, *** *p* < 0.001 vs. control. Scale bar denotes 5 μm.

To determine whether *P. cuspidatum*-derived extracts exert anticancer effects through modulation of inflammation and oxidative stress—mechanisms attributed to resveratrol—we examined the effects of PE and its microbially transformed counterpart, CPE, on inflammatory signaling and intracellular ROS levels in A549 cells (Figure 3). Both PE and CPE significantly suppressed the expression of inflammation-associated proteins, including NLRP3, pro-caspase-1, NF-κB, IL-6, and TNF-α, in a dose-dependent manner. PE preferentially reduced downstream pro-inflammatory cytokines, particularly TNF-α, consistent with reported anti-inflammatory actions of resveratrol and polydatin (Figure 3A). In contrast, CPE more strongly inhibited upstream inflammasome components NLRP3 and pro-caspase-1, suggesting enhanced interference with inflammasome activation [28] following microbial transformation (Figure 3B).

Given the close interplay between oxidative stress and inflammatory signaling, intracellular ROS levels were also assessed by DCFDA (Figure 3C). PE treatment reduced ROS accumulation at lower concentrations, supporting its antioxidant role in suppressing NF-κB and inflammasome-mediated inflammation. In contrast, CPE induced a dose-dependent increase in ROS at higher concentrations, indicating a shift toward pro-oxidant stress that may contribute to apoptosis in cancer cells, consistent with resveratrol-associated anticancer pathways that regulate inflammation and oxidative stress.

### 3.4. PE and CPE Induce Apoptosis in A549 Cells via Differential Regulation of Apoptotic Pathways

**Figure 4:**
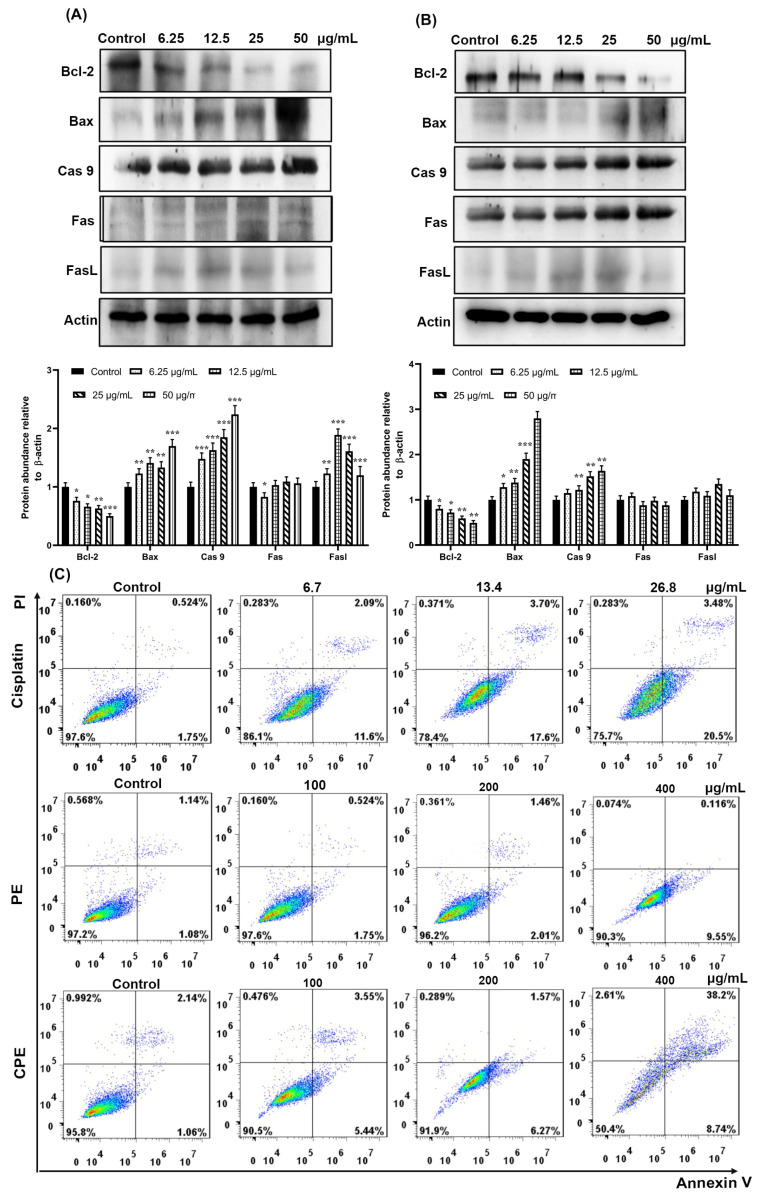
PE and CPE induce apoptosis in A549 cells through distinct apoptotic pathways. A549 cells were treated with PE or CPE for 24 h and analyzed by Western blotting and Annexin V/PI flow cytometry. (**A**) PE decreased Bcl-2 and increased Bax, caspase-9, Fas, and FasL, suggesting activation of both intrinsic and partial extrinsic apoptotic pathways. (**B**) CPE markedly reduced Bcl-2 and increased Bax and caspase-9, with minimal effects on Fas/FasL, indicating predominant activation of mitochondrial apoptosis. (**C**) Annexin V/PI analysis confirmed apoptosis induction at 72 h. PE moderately increased total apoptosis to 9.67% at 400 μg/mL, whereas CPE strongly increased apoptosis to 46.94%, mainly through late apoptosis/PI-positive cells. Cisplatin served as a positive control. Data are mean ± SEM (*n* = 3). * *p* < 0.05, ** *p* < 0.01, *** *p* < 0.001 vs. control.

In the DCFDA assay, PE and CPE induced ROS accumulation. This investigation also examined whether oxidative stress was associated with activation of apoptotic signaling in A549 cells. Western blot analysis revealed that both PE and CPE significantly modulated key apoptosis-related proteins after 24 h of treatment (Figure 4). PE treatment induced a dose-dependent decrease in the anti-apoptotic protein Bcl-2, accompanied by increased expression of the pro-apoptotic protein Bax and caspase-9. This resulted in a marked elevation of the Bax/Bcl-2 ratio, indicating activation of the mitochondrial apoptotic pathway (Figure 4A). In addition, PE upregulated Fas and FasL, suggesting partial engagement of extrinsic apoptotic signaling.

CPE elicited a distinct but related response; Bcl-2 levels were similarly reduced, and Bax expression was more strongly upregulated than with PE, leading to a pronounced increase in the Bax/Bcl-2 ratio (Figure 4B). Caspase-9 levels were also elevated, supporting robust activation of mitochondrial apoptosis. In contrast to PE, CPE had minimal effects on Fas and FasL expression, indicating that CPE-induced apoptosis is predominantly mediated through the intrinsic pathway, whereas PE activates both intrinsic and extrinsic components.

To validate whether the changes in apoptosis-related proteins reflected functional apoptosis, Annexin V/PI flow cytometry was performed on A549 cells (Figure 4C). Cisplatin confirmed assay sensitivity, increasing total apoptotic cells 23.98% at 26.8 μg/mL. PE induced a weaker apoptotic response, with total apoptosis increasing from 2.22% in control to 2.27%, 3.47%, and 9.67% at 100, 200, and 400 μg/mL, mainly due to increased early apoptosis at 400 μg/mL (9.55%). In contrast, CPE showed a markedly stronger effect, increasing total apoptosis from 3.20% in the control to 8.99%, 7.84%, and 46.94% at 100, 200, and 400 μg/mL, respectively. Notably, 400 μg/mL CPE caused a pronounced increase in the percentage of late apoptotic cells (38.2%). This indicates that CPE not only initiates apoptosis but also promotes progression toward late-stage apoptotic cell death. These results are consistent with Figure 4A,B, supporting the idea that PE induces moderate apoptosis through intrinsic and partial extrinsic signaling, whereas CPE predominantly triggers robust mitochondrial apoptosis through Bcl-2 downregulation and Bax/caspase-9 activation.

### 3.5. In Vivo Antitumor Effects of PE and CPE in the A549 Xenograft Model

**Figure 5:**
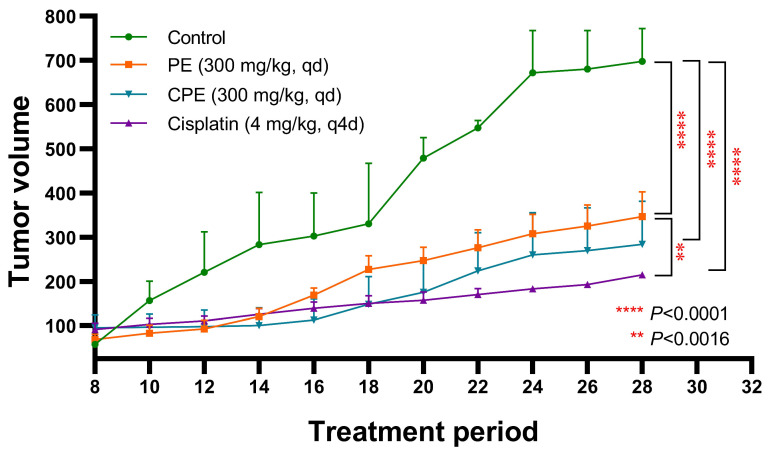
In vivo antitumor effects of PE and CPE in the A549 xenograft model. BALB/c nude mice bearing A549 tumors (~100 mm^3^) were treated once daily with vehicle, PE, CPE, or cisplatin for 20 days. Tumor volumes were measured every two days, normalized to Day 8, and plotted over time. Vehicle-treated tumors grew rapidly, while PE and CPE reduced tumor growth, with CPE showing greater suppression than PE. Cisplatin served as a positive control and produced the strongest inhibition. Data are presented as mean tumor volume over time. Data are mean ± SEM (*n* = 5). ** *p* < 0.0016, **** *p* < 0.0001 vs. control.

To determine whether the ROS-associated, pro-apoptotic activity observed in vitro translates into antitumor efficacy in vivo, we evaluated PE and CPE in an NSCLC A549 xenograft model (Figure 5). Mice bearing established A549 tumors received PE or CPE (300 mg/kg, p.o., qd) once daily for 20 consecutive days; cisplatin (4 mg/kg, i.p., q4d for five doses) was used as a positive control, and saline as the vehicle control. Both PE and CPE significantly suppressed tumor growth compared with vehicle, resulting in substantially reduced endpoint tumor volumes (~350 mm^3^ for PE and ~290 mm^3^ for CPE versus ~700 mm^3^ for vehicle; both *p* < 0.0001). These endpoint values correspond to an estimated tumor growth suppression of ~50.0% for PE and ~58.6% for CPE relative to vehicle. In addition, CPE produced significantly greater tumor growth inhibition than PE (*p* = 0.0016), indicating enhanced in vivo activity following microbial conversion. Cisplatin yielded the greatest antitumor effect, reducing endpoint tumor volume to ~210 mm^3^ (~70.0% suppression versus vehicle; *p* < 0.0001). Overall, oral administration of CPE exerted a stronger antitumor effect than PE in the A549 xenograft model, consistent with enhanced anticancer efficacy after microbial biotransformation and concordant with the ROS-associated apoptotic responses observed in vitro.

### 3.6. Subacute Oral Toxicity of Resveratrol-Enriched CPE

To evaluate the subacute safety of the resveratrol-enriched fermented CPE, a 28-day repeated oral toxicity study was conducted in male rats. Animals received daily doses of CPE at 0 (control), 167, or 500 mg/kg, and hematological and plasma biochemical parameters were assessed at the end of the treatment period to identify potential treatment-related toxic effects.

For the hematological parameters, as shown in Table 1, no statistically significant or dose-dependent changes were observed in major hematological indices following CPE administration. Erythrocyte count, hemoglobin concentration, hematocrit, and red blood cell indices (MCV, MCH, and MCHC) remained comparable between control and treated groups, indicating that CPE did not impair erythropoiesis or oxygen-carrying capacity. Platelet counts and total leukocyte numbers were also unaffected, and the proportions of lymphocytes and segmented neutrophils remained within normal physiological ranges. Prothrombin time showed no consistent dose-related alterations, suggesting that CPE did not affect coagulation function. Overall, the hematological profile indicates the absence of bone marrow toxicity, anemia, immune dysregulation, or coagulation abnormalities following 28 days of oral CPE exposure.

The plasma biochemical parameters in Table 2 further support the lack of systemic toxicity. Liver function markers, including AST, ALT, ALP, and γ-GT, showed no dose-dependent increases across treatment groups, indicating that CPE did not induce hepatocellular injury or cholestasis. Total protein, albumin, and globulin levels remained stable, suggesting preserved hepatic synthetic function.

Renal function markers, including blood urea nitrogen (BUN) and creatinine, were not significantly altered by CPE treatment, demonstrating the absence of nephrotoxicity even at the highest dose tested. In addition, glucose and total bilirubin levels were comparable between control and treated animals, indicating no disruption of carbohydrate metabolism or bilirubin clearance.

Toxicological assessment and NOAEL determination, as well as hematological and biochemical data, demonstrate that repeated oral administration of a resveratrol-enriched CPE for 28 days does not induce toxicologically relevant adverse effects in male rats at doses up to 500 mg/kg/day. Importantly, no evidence of liver, kidney, hematopoietic, or metabolic toxicity was observed, despite the extract’s high resveratrol content (~70%), supporting the toxicological safety of CPE and providing a critical foundation for its further development as a functional food ingredient or therapeutic agent.

### 3.7. Histopathological Assessment of Organ Toxicity After High-Dose CPE in Rats

**Figure 6:**
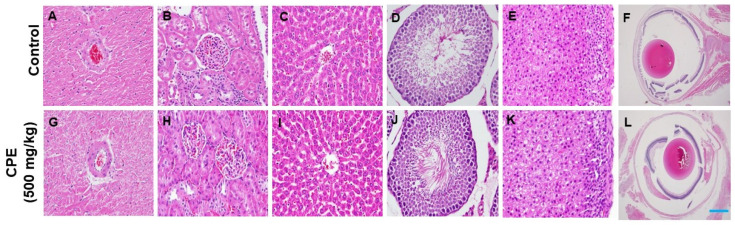
Histopathological evaluation of major organs following high-dose CPE administration in rats. Representative H&E-stained sections of heart (**A**,**G**), kidney (**B**,**H**), liver (**C**,**I**), spleen (**D**,**J**), testes (**E**,**K**), adrenal gland (**F**,**L**) (400×), and eye (20×) from control and CPE high-dose (500 mg/kg/day, 28 days) groups are shown. No significant histopathological changes are observed in any organ between control and treated animals, indicating the absence of CPE-related tissue toxicity. Scale bar denotes 5 μm.

Rats administered resveratrol-enriched CPE at 500 mg/kg/day for 28 days showed no microscopic evidence of organ toxicity. Histopathological examination of H&E-stained sections from the heart, kidney, liver, spleen, testes, adrenal glands, and eyes revealed normal tissue architecture without treatment-related lesions (Figure 6). The myocardium displayed preserved fiber alignment, renal glomeruli and tubules were intact, and hepatic lobular structure appeared normal with no necrosis or inflammatory cell infiltration. Splenic white and red pulp were well maintained without lymphoid depletion, testicular sections exhibited normal seminiferous tubules with ongoing spermatogenesis, and the adrenal cortex and medulla showed no degenerative changes. Ocular tissues, including retinal and corneal structures, were also unremarkable. These findings indicate that repeated high-dose CPE administration does not induce detectable histopathological alterations in major organs, supporting its systemic safety profile for further development.

## 4. Discussion

Comprehensive evidence that microbial transformation represents a promising biotechnological strategy to enhance both the efficacy and safety profile of traditional herbal medicines for cancer therapy has been reported [29,30]. By directly comparing native PE with CPE across phytochemical, cellular, animal, and toxicological platforms, we demonstrate that microbial biotransformation reshapes the chemical profile in a functionally relevant manner and results in enhanced antitumor activity without additional toxicity, consistent with prior reports showing that fermentation-based transformation improves the bioactivity and safety of polyphenol-rich herbal extracts [31,32]. Collectively, these findings address key limitations of conventional herbal extracts and support further development of CPE for NSCLC.

Quantitative analysis revealed that microbial transformation in our system markedly altered the phytochemical composition of *P. cuspidatum* extract. Most notably, polydatin was efficiently converted to resveratrol, yielding a 2.7-fold increase in resveratrol content with near-complete depletion of polydatin. This pattern is consistent with β-glucosidase-mediated hydrolysis of glycosidic bonds and with the inherently low abundance of resveratrol in native *P. cuspidatum*. This shift is pharmacologically relevant; compared with polydatin, resveratrol shows greater cellular uptake and bioavailability due to its aglycone structure, enabling stronger interactions with intracellular target [10,19,21]. In parallel, the modest reduction in emodin, a compound associated with hepatotoxicity at high doses, further improves the safety-to-efficacy balance of the transformed extract [33]. Unlike conventional extraction approaches that proportionally concentrate all constituents, microbial biotransformation enables selective enrichment of pharmacologically beneficial compounds while limiting the accumulation of potentially adverse components [19,24]. Fermentation likely alters the entire phytochemical matrix. The greater activity of CPE versus PE suggests effects beyond resveratrol, reflecting coordinated changes in overall composition.

The optimized phytochemical profile of CPE was associated with significantly stronger antiproliferative effects, preventing tumor growth, metastasis to the lung, and tumor-induced neovascularization in Lewis lung carcinoma [34]. CPE reduced A549 cell viability in a dose-dependent manner with lower IC_50_ values than PE, while sparing normal lung epithelial cells at equivalent concentrations. This cancer-selective activity indicates a favorable therapeutic window, a critical consideration for translational relevance. CPE also exerted pronounced antimetastatic effects, as evidenced by wound-healing assays. Given that metastasis accounts for most NSCLC-related mortality, agents that inhibit both tumor growth and cell migration are of particular interest. The superior inhibition of migration by CPE suggests modulation of pathways involved in cell adhesion and extracellular matrix remodeling.

Mechanistic evidence indicates that CPE suppresses NSCLC progression by coordinating the regulation of apoptotic and inflammatory signaling networks rather than through a single cytotoxic mechanism. The increased Bax/Bcl-2 ratio, together with caspase-9 activation, demonstrates the restoration of mitochondrial apoptotic competence, a pathway frequently impaired in lung cancer cells due to elevated anti-apoptotic proteins and defective mitochondrial permeability control [34,35,36]. Reinstating this balance is a decisive step that commits cells to programmed death and limits their ability to recover from stress [37,38]. Together, these findings indicate that PE and CPE induce apoptosis in A549 cells through overlapping but mechanistically distinct pathways. The Western blot results showed that both extracts reduced the anti-apoptotic protein Bcl-2 and increased Bax and caspase-9 expression, supporting activation of the intrinsic mitochondrial apoptotic pathway. However, PE additionally increased Fas and FasL expression, suggesting that PE may partially engage extrinsic apoptotic signaling. In contrast, CPE had minimal effects on Fas/FasL but induced a stronger increase in Bax and caspase-9, indicating that CPE-mediated apoptosis is predominantly mitochondria-dependent. This mechanistic difference was further supported by Annexin V/PI flow cytometry. PE induced only a moderate increase in apoptosis, reaching 9.67% total apoptotic cells at 400 μg/mL, whereas CPE markedly increased total apoptosis to 46.94% at the same concentration, mainly through an increase in late apoptotic/PI-positive cells. These results suggest that microbial conversion enhances the pro-apoptotic activity of Polygonum cuspidatum extract and shifts its anticancer effect toward a more robust intrinsic apoptotic response. In line with the ROS accumulation observed in the DCFDA assay, CPE-induced oxidative stress may contribute to mitochondrial dysfunction, Bax/Bcl-2 imbalance, caspase-9 activation, and subsequent apoptotic progression in A549 cells.

The apoptotic profile induced by CPE is broadly consistent with previously reported mechanisms of resveratrol in NSCLC cells. Resveratrol has been shown to induce apoptosis in A549 lung adenocarcinoma cells through mitochondrial and p53-associated signaling, accompanied by increased Bax expression, reduced Bcl-2 expression, and activation of apoptotic cell death. In agreement with these reports, CPE markedly increased ROS accumulation, reduced Bcl-2, enhanced Bax and caspase-9 expression, and promoted Annexin V/PI-positive apoptotic cell death, particularly late apoptosis at the highest concentration [39]. These findings suggest that microbial conversion may enhance resveratrol-associated pro-apoptotic activity in Polygonum cuspidatum extract and shift the response toward a predominantly mitochondria-dependent apoptotic mechanism. However, because resveratrol has also been reported to regulate autophagy–apoptosis crosstalk and Fas/caveolin-1-associated signaling in A549 cells, further studies examining p53, Akt/MDM2, mitochondrial membrane potential, cytochrome c release, caspase-3/PARP cleavage, and autophagy markers are required to determine whether CPE fully recapitulates the canonical resveratrol-mediated apoptotic pathway or exerts additional extract-specific effects [40].

At the same time, CPE markedly inhibited NLRP3 inflammasome components and NF-κB signaling, two pathways that sustain tumor-associated inflammation and promote proliferation, angiogenesis, and resistance to therapy [41]. These pathways normally promote survival by inducing cytokines such as IL-6 and TNF-α and by upregulating anti-apoptotic genes [42,43]. Their suppression suggests that CPE weakens protective inflammatory signaling that would otherwise counteract apoptosis. The simultaneous modulation of mitochondrial death signaling and inflammatory survival pathways, therefore, represents a complementary mechanism that restricts adaptive resistance. Importantly, CPE did not reduce intracellular ROS levels, indicating that its anticancer effects are unlikely to arise from antioxidant activity, which can, in some contexts, protect malignant cells from oxidative damage. This interpretation is consistent with reports that resveratrol promotes cancer cell apoptosis through ROS-dependent signaling and mitochondrial pathway regulation rather than cytoprotective antioxidant effects [44,45]. Instead, CPE appears to act by directly modulating signaling proteins that govern cell-fate decisions, supporting a model in which microbial transformation enhances extract potency and shifts tumor cells from a pro-survival, inflammatory state toward apoptotic elimination through coordinated pathway control.

Further xenograft studies confirmed that the enhanced in vitro activity of CPE translates into significant tumor growth inhibition in vivo. Oral administration of CPE produced tumor suppression comparable to PE, with no adverse effects on body weight or behavior. The oral route is clinically relevant and supports feasibility for long-term administration. Despite its higher resveratrol content, CPE did not show proportionally greater in vivo efficacy at equivalent doses, suggesting that pharmacokinetics, metabolism, or matrix effects influence systemic activity [46,47,48]. These observations highlight the need for pharmacokinetic studies to define exposure–response relationships and optimize dosing strategies. This CPE’s mechanism of action suggests potential compatibility with existing NSCLC therapies; it targets apoptosis and inflammation rather than DNA synthesis or specific oncogenic drivers. CPE may complement chemotherapy, targeted therapy, or immunotherapy without overlapping toxicity. Combination studies are, therefore, a logical next step.

The 28-day repeated-dose toxicity study demonstrated that CPE is well tolerated, with no evidence of hepatic, renal, hematological, or histopathological toxicity even at doses five times higher than those used in efficacy studies. This establishes a no-observed-adverse-effect level of at least 500 mg/kg/day. The absence of liver and kidney toxicity is notable given concerns surrounding emodin and high-dose purified resveratrol. The favorable safety profile of CPE likely reflects the protective effects of the complex phytochemical matrix and fermentation-derived metabolites [49,50,51]. Such matrix effects may influence metabolism, reduce peak systemic exposure, or counteract toxicity, consistent with established principles of natural product pharmacology.

## 5. Conclusions

Beyond *P. cuspidatum*, this work supports microbial biotransformation as a generalizable strategy for improving traditional herbal medicines. Many medicinal plants contain glycosylated precursors with limited bioavailability. Fermentation provides a scalable, environmentally sustainable method to enhance bioactivity while preserving the complexity of natural extracts [52,53,54]. By integrating traditional botanical knowledge with modern biotechnology, microbial transformation offers a practical route toward evidence-based optimization of herbal therapeutics. This approach may be particularly valuable for applications where long-term safety, oral delivery, and multi-target activity are desired.

## Figures and Tables

**Table 1:** Hematological Analysis in a 28-Day Oral Toxicity Study with Male Rats.

Parameters	Control	167 mg/kg	500 mg/kg
Erythrocytes (10^6^/L)	8.7 ± 0.9	9.0 ± 0.7	8.9 ± 0.9
Hemoglobin (g/dL)	18.7 ± 2.0	19.1 ± 1.4	18.7 ± 2.0
Hematocrit (%)	54.1 ± 5.4	55.6 ± 4.1	55.1 ± 5.2
MCV (fL)	61.9 ± 1.5	61.5 ± 2.0	62.2 ± 1.8
MCH (pg)	21.4 ± 0.7	21.2 ± 0.8	21.1 ± 0.8
MCHC (g/dL)	34.5 ± 0.6	34.4 ± 0.7	33.9 ± 0.8
Platelets (10^3^/L)	990 ± 248	1031 ± 116	1011 ± 127
Leukocytes (10^3^/L)	9.7 ± 1.8	10.7 ± 3.4	10.2 ± 2.2
Lymphocytes (%)	86.4 ± 2.9	88.2 ± 2.9	83.8 ± 5.8
Seg. Neu (%)	8.3 ± 1.5	9.0 ± 2.5	8.5 ± 1.6
PT (s)	10.4 ± 0.2	10.6 ± 0.2	12.2 ± 3.2

All values are means S.D. (*n* = 10). M: male; Seg. Neu: segmented neutrophil.

**Table 2:** Plasma Biochemical Examination in a 28-Day Oral Toxicity Study in Rats.

Parameters	Control	167 mg/kg	500 mg/kg
ALP (U/L)	130.4 ± 6.8	114.3 ± 10.0	111.9 ± 14.3
AST (U/L)	78.3 ± 7.7	73.5 ± 7.8	72.1 ± 2.4
ALT (U/L)	45.3 ± 2.3	42.6 ± 2.9	49.6 ± 2.4
γ-GT (IU/L)	3.1 ± 1.9	4.1 ± 2.1	4.8 ± 2.7
Total-protein (g/dL)	5.7 ± 0.2	5.7 ± 0.1	5.8 ± 0.2
Albumin (g/dL)	3.5 ± 0.0	3.5 ± 0.1	3.5 ± 0.0
Globulin (g/dL)	2.2 ± 0.1	2.1 ± 0.2	2.3 ± 0.2
T-Bilirubin (mg/dL)	1.34 ± 0.03	1.39 ± 0.04	1.42 ± 0.02
Glucose (mg/dL)	101.5 ± 26.3	112.1 ± 25.0	106.8 ± 21.0
BUN (mg/dL)	14.2 ± 0.7	14.7 ± 0.8	12.5 ± 1.1
Creatinine (mg/dL)	0.60 ± 0.03	0.58 ± 0.01	0.68 ± 0.03

## Data Availability

The data supporting the findings of this study are available from the corresponding authors upon reasonable request. If deposition in a public repository is required at submission or revision, the repository information and persistent identifier will be added in the final version.

## Supplementary Materials

The following supporting information can be downloaded at: https://www.mdpi.com/article/10.3390/nu18121862/s1, Figure S1: PDA-based confirmation of resveratrol in bioconverted *Polygonum cuspidatum* extract, Table S1: List of primary antibodies for Western blot.
