# Supplementary material for: Resveratrol-Enriched Polygonum cuspidatum Extract Enhances Functional Bioactivity Against Non-Small Cell Lung Cancer Through Modulation of Inflammatory Signaling and Mitochondrial Apoptosis
Source: Nutrients. 2026 Jun 9;18(12):1862. doi: 10.3390/nu18121862 (PMC13304522; doi:10.3390/nu18121862)
Supplement: Supplementary file 1 [file nutrients-18-01862-s001.zip › nutrients-4268963-supplementary.pdf]

Supplementary data

Resveratrol-enriched *Polygonum cuspidatum* extract enhances functional bioactivity against non-small cell lung cancer through modulation of inflammatory signaling and mitochondrial apoptosis

Ho-Lin Wang [a,†], Hui-Pei Huang [b,c,†], Naveen Ranasinghe [d,†], Yu-Hsien Lin [e], Hsiao-Ping Kuo [f], Shyue-Tsong Huang [f], Li-Chan Yang [d], Tai-Lin Chen [d,g,*] and Ming-Hon Hou [a,h,i,*]

[a] Doctoral Program in Medical Biotechnology, National Chung Hsing University, Taichung 402, Taiwan
[b] Department of Medical Research, Chung Shan Medical University Hospital, Taichung 402, Taiwan
[c] Department of Biochemistry, School of Medicine, Chung Shan Medical University, Taichung 402, Taiwan
[d] Department of Post-Baccalaureate Medicine, College of Medicine, National Chung Hsing University, Taichung 402, Taiwan
[e] Doctoral Program in Tissue Engineering and Regenerative Medicine, National Chung Hsing University, Taichung 402, Taiwan
[f] Bioresources Collection and Research Center, Food Industry Research and Development Institute, Hsinchu 30062, Taiwan
[g] Graduate Institute of Chinese Medicine and Drug Development, School of Medicine, National Chung Hsing University, Taichung 402, Taiwan
[h] Graduate Institute of Genomics and Bioinformatics, National Chung Hsing University, Taichung 402, Taiwan
[i] Biotechnology Center, National Chung Hsing University, Taichung 402, Taiwan

[†] These authors contributed equally to this work.
* Correspondence: Tai-Lin Chen (chentl@nchu.edu.tw; Tel: +886 4 2284 0360 ext. 912) and Ming-Hon Hou (mhho@nchu.edu.tw; Tel: +886 4 2284 0338 ext. 7011).

Supplementary data

PDA-based confirmation of resveratrol in bioconverted *Polygonum cuspidatum* extract.

HPLC-PDA analysis was performed to verify the peak identity of resveratrol in the bioconverted *P. cuspidatum* extract. The authentic resveratrol standard showed a major peak at a retention time of approximately 18.203 min, with characteristic UV absorption maxima at approximately 216.2 and 305.2 nm. The bioconverted *P. cuspidatum* extract displayed a corresponding major peak at approximately 18.249 min. The PDA-derived UV absorption spectrum of this sample peak showed absorption maxima at approximately 216.2 and 305.2 nm, closely matching those of the authentic resveratrol standard. These results support the assignment of the major HPLC peak in the bioconverted extract as resveratrol based on retention-time matching and UV spectral concordance. Quantitative analysis indicated that the resveratrol content in the bioconverted extract was 547.84 ± 9.70 mg/g.

Because peak overlap can occur in HPLC-based analysis, the present PDA data provide an additional orthogonal UV spectral comparison to support peak assignment. However, the peak identity was not confirmed by LC-MS or ^{1}H NMR in the current study. This limitation has been clarified in the revised supplementary information.

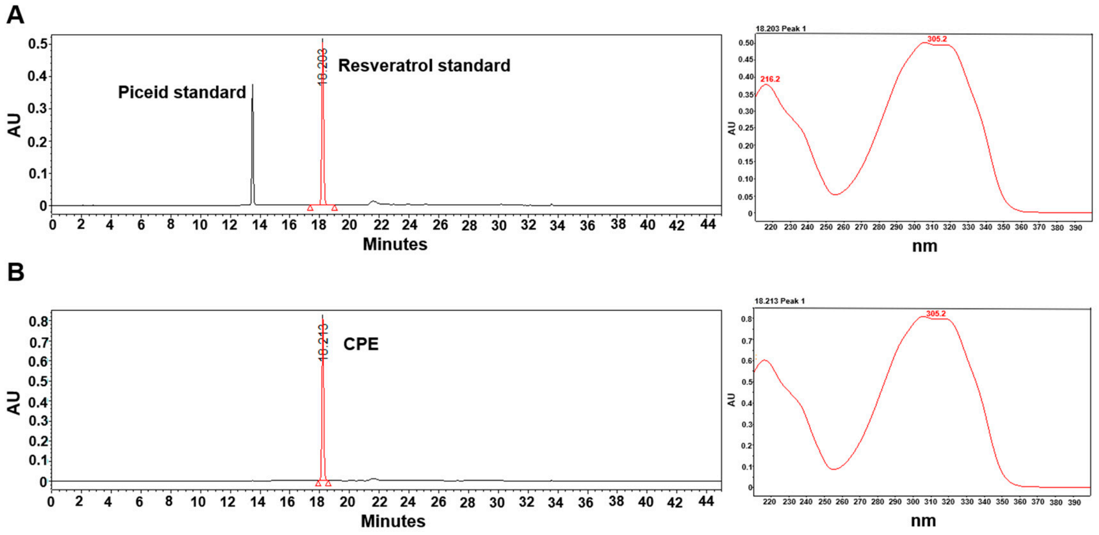

Figure S1. HPLC-PDA confirmation of resveratrol in bioconverted *Polygonum cuspidatum* extract. (A) bioconverted *P. cuspidatum* extract (B). The major peak in the bioconverted extract showed a retention time and UV absorption spectrum comparable to those of the resveratrol standard.

Table S1. List of primary antibodies for Western blot.

Type	Target	CST antibody (clone)	Catalog No.	Host	Suggested WB dilution	Manufacturer
Primary	NLRP3	NLRP3 (D2P5E) Rabbit mAb	13158	Rabbit	1:1000	Cell Signaling
Primary	Pro-/cleaved Caspase-1	Caspase-1 Antibody	2225	Rabbit	1:1000	Cell Signaling
Primary	NF-κB p65	NF-κB p65 (L8F6) Mouse mAb	6956	Mouse	1:1000	Cell Signaling
Primary	IL-6	IL-6 (D3K2N) Rabbit mAb (human)	12153	Rabbit	1:1000	Cell Signaling
Primary	TNF-α	TNF-α Antibody	3707	Rabbit	1:1000	Cell Signaling
Primary	β-Actin	β-Actin Antibody	4967	Rabbit	1:1000	Cell Signaling
Primary	Bcl-2	Bcl-2 Antibody	2876	Rabbit	1:1000	Cell Signaling
Primary	Bax	Bax Antibody	2772	Rabbit	1:1000	Cell Signaling
Primary	Caspase-9	Caspase-9 Antibody (human)	9502	Rabbit	1:1000	Cell Signaling
Primary	Fas (CD95)	Fas (C18C12) Rabbit mAb	4233	Rabbit	1:1000	Cell Signaling
Primary	FasL (CD95L)	FasL (D1N5E) Rabbit mAb (human)	68405	Rabbit	1:1000	Cell Signaling
